# Patient Satisfaction and Hospital Quality of Care Evaluation in Malaysia Using SERVQUAL and Facebook

**DOI:** 10.3390/healthcare9101369

**Published:** 2021-10-14

**Authors:** Afiq Izzudin A. Rahim, Mohd Ismail Ibrahim, Kamarul Imran Musa, Sook-Ling Chua, Najib Majdi Yaacob

**Affiliations:** 1 Department of Community Medicine, School of Medical Science, Universiti Sains Malaysia, Kubang Kerian, Kota Bharu 16150, Kelantan, Malaysia; drafiqrahim@student.usm.my (A.I.A.R.); drkamarul@usm.my (K.I.M.); 2 Faculty of Computing and Informatics, Multimedia University, Persiaran Multimedia, Cyberjaya 63100, Selangor, Malaysia; slchua@mmu.edu.my; 3 Unit of Biostatistics and Research Methodology, Health Campus, School of Medical Sciences, Universiti Sains Malaysia, Kubang Kerian, Kota Bharu 16150, Kelantan, Malaysia; najibmy@usm.my

**Keywords:** patient satisfaction, service quality, SERVQUAL, Facebook, machine learning, patient online review, Malaysia

## Abstract

Social media sites, dubbed patient online reviews (POR), have been proposed as new methods for assessing patient satisfaction and monitoring quality of care. However, the unstructured nature of POR data derived from social media creates a number of challenges. The objectives of this research were to identify service quality (SERVQUAL) dimensions automatically from hospital Facebook reviews using a machine learning classifier, and to examine their associations with patient dissatisfaction. From January 2017 to December 2019, empirical research was conducted in which POR were gathered from the official Facebook page of Malaysian public hospitals. To find SERVQUAL dimensions in POR, a machine learning topic classification utilising supervised learning was developed, and this study’s objective was established using logistic regression analysis. It was discovered that 73.5% of patients were satisfied with the public hospital service, whereas 26.5% were dissatisfied. SERVQUAL dimensions identified were 13.2% reviews of tangible, 68.9% of reliability, 6.8% of responsiveness, 19.5% of assurance, and 64.3% of empathy. After controlling for hospital variables, all SERVQUAL dimensions except tangible and assurance were shown to be significantly related with patient dissatisfaction (reliability, *p* < 0.001; responsiveness, *p* = 0.016; and empathy, *p* < 0.001). Rural hospitals had a higher probability of patient dissatisfaction (*p* < 0.001). Therefore, POR, assisted by machine learning technologies, provided a pragmatic and feasible way for capturing patient perceptions of care quality and supplementing conventional patient satisfaction surveys. The findings offer critical information that will assist healthcare authorities in capitalising on POR by monitoring and evaluating the quality of services in real time.

## 1. Introduction

The World Health Organization (WHO) stresses that substandard care wastes significant resources and jeopardises public health by degrading human capital and decreasing productivity. Thus, in addition to providing effective coverage of essential health services and financial security in each country, delivering high-quality care or service is important in achieving the Universal Health Coverage goal [1]. At the core of delivering high-quality care is a dedication to person-centered care. Communities must be engaged in the design, implementation, and ongoing evaluation of health services to ensure that they meet local health needs. Also, striking a balance between patient expectations and quality improvement initiatives is important, since it influences patient safety, survival, and long-term health [2]. According to a systematic analysis, poor healthcare quality was the main factor leading to an increase in deaths from cardiovascular disease, neonatal trauma, and communicable illnesses [3]. As healthcare prepares for the Industrial Revolution 4.0 by becoming more patient-centered and value-driven, quality management systems must include efforts to understand and respect patients’ interests, desires, and values. Because such reports can only be generated by patients, it is critical to create systems for monitoring patient experiences and to promote their use on an individual and communal level [4,5]. Patient perception and satisfaction have been a key component of patient-centered care since the early 1990s and have been incorporated into healthcare quality of care assessment. Healthcare administrators that aim for excellence consider patient perception while creating strategies for improving treatment quality [6].

Service quality (SERVQUAL) is a commonly used technique for evaluating the quality of service in a wide variety of service environments, sectors, and nations [7]. Because the model encompasses five dimensions—tangible, reliability, responsiveness, empathy, and assurance—it efficiently measures customer service needs and perceptions [8].

SERVQUAL, Hospital Consumer Assessment of Healthcare Providers and Systems (HCAHPS), and other traditional patient satisfaction surveys are the product of years of evaluative analysis, are performed and evaluated in a methodical manner, and may evoke a wide variety of answers from patients [9,10]. However, traditional patient or public surveys used to assess the quality of healthcare services are time and resource intensive, require considerable time between hospital admission and report disclosure, frequently result in a failure to identify the underlying causes of concern, and introduce response and selection bias [11,12]. The disconnect between conventional surveys and patient perceptions and treatment quality underscored the need for developing new data sources for assessing patient perceptions and care quality [13]. Technological innovation is essential for creating new ways for rapidly assessing the quality of services at an affordable cost. Therefore, social media platforms, which are often referred to as patient online reviews (POR), have been suggested as a new way for gauging patient satisfaction and monitoring treatment quality [14,15].

There have been small number of POR studies in contrast to its exponential growth [16,17]. While it has been demonstrated that Facebook and other social media platforms can improve health outcomes through health education and information [18,19] and can be beneficial during public health crises [20,21], other studies have examined specific features of social media platforms such as reviews and ratings and their relationship to patient satisfaction and hospital quality measures [16]. For example, Facebook offers a review feature that allows users to leave narrative assessments and evaluate the performance of companies and institutions on their Facebook pages. Numerous studies have discovered a weak to moderate correlation between Facebook evaluations and traditional patient satisfaction survey metrics [22,23,24,25], while another study discovered a link between clinical quality indicators such as reduced re-admission rates and higher Facebook ratings [26]. According to recent research, hospitals with an active Facebook page had a higher number of “likes,” a greater percentage of patients ready to refer the hospital, and a higher overall satisfaction score [27]. Additional study on the patient viewpoint and its relationship to hospital patients’ total Facebook ratings discovered associations with a variety of issues, including wait times, treatment effectiveness, and communication [28]. With an increasing number of patients asking and freely sharing hospital evaluations on social media, feedback data may supplement conventional patient satisfaction surveys [14,27].

However, the unstructured nature of POR data collected from social media presents several difficulties, including data cleaning and processing. While this may be accomplished manually via human input, the process is lengthy, and the method’s validity and reliability are often questioned [29]. A systematic evaluation of POR was proposed to accelerate the processing of large-scale online data review using sophisticated analytical techniques such as machine learning [16]. Consequently, a machine learning approach for classifying service quality themes or subjects based on unstructured social media data has the potential to significantly improve healthcare quality of care [30,31].

Additionally, the population’s fondness for social media has led many healthcare institutions to use their country’s most popular social media platforms for online communication and engagement with the public. According to a national survey conducted in Taiwan, Facebook has a high level of penetration and popularity in the country, which may be one of the reasons why more than half of Taiwan’s hospitals have established an official Facebook profile [32]. Facebook is also a critical component of Malaysian social media use. According to a 2020 survey, 91.7 percent of Malaysian internet users utilised Facebook, and the site is projected to continue to be the country’s most popular social networking site [33]. Given the popularity of Facebook in Malaysia and its expanding usage in healthcare, this study’s first task was to assess the frequency of SERVQUAL dimensions in Facebook reviews of Malaysian public hospitals using a machine learning classifier and prevalence of hospital patient satisfaction. The second was to seek to establish relationships between SERVQUAL qualities and hospital patient dissatisfaction as expressed in Facebook reviews. POR analyzed using a machine learning algorithm may have value in assisting all key healthcare stakeholders in making decisions to enhance the quality of care delivered in Malaysia.

## 2. Related Work

### 2.1. Patient Satisfaction

Intellectuals have been assessing hospital patient satisfaction for years, using a range of methodologies and conceptual frameworks. An earlier study showed that patients with moderate expectations reported the highest levels of satisfaction, whereas those with excessive expectations reported the lowest levels of satisfaction [34]. When patients’ expectations were met in terms of health care delivery, they reported satisfaction with such services [35]. Since those early attempts, the number of factors linked with patient satisfaction have increased dramatically and vary between research [36,37]. However, one systematic review found that two significant determinants of patient satisfaction were variables affecting the healthcare provider and patient characteristics [35]. Across studies, that study found that provider-related variables were the strongest predictor of patient satisfaction. There were nine identified determinants of healthcare services: technical care, interpersonal care, physical environment, accessibility, availability, financial resources, organisational characteristics, continuity of treatment, and care result. Research that examined the physical environment in relation to patient satisfaction ratings on social media discovered that environmental variables such as parking, cleanliness, and waiting rooms all contributed to patient satisfaction [38]. Another POR research showed that comments on the efficacy of treatment, communication, and diagnostic quality were most strongly linked with patients’ overall ratings [28]. A comprehensive assessment of patient satisfaction confirmed the results, revealing that interpersonal skills and technical care features had the most positive associations with service-related factors [35].

Patient characteristics such as age, gender, education, socioeconomic status, marital status, race, religion, geographic characteristics, frequency of visits, length of stay, health status, personality, and expectations were all investigated to ascertain their associations with patient satisfaction [35]. Hospital characteristics such as location and rural regions were shown to be positively associated with patient discontent [39], even though another study found rural residents were satisfied with healthcare services [40]. Additionally, the size and type of hospital services influenced patient satisfaction [15,41]. Previously, it was believed that people would be more unhappy with a service that dealt with a greater number of patients and a bigger office. However, in a comprehensive assessment of patient satisfaction, these associations were modest and inconsistent [35]. Therefore, the research concluded that it may be worthwhile to attempt to build patient satisfaction using health care quality indicators and observe how individuals increase their satisfaction with health services. SERVQUAL and HCAHPS are two examples of systematic surveys that assess healthcare quality of care. The findings of patient satisfaction surveys may be very helpful for both healthcare professionals and patients. They aid healthcare providers in finding areas in which their services might be improved. Increased patient satisfaction with healthcare services boosts public hospital responsiveness [42]. Additionally, it enables policymakers to understand patient needs and therefore create strategic plans for more effective and high-quality services. According to studies, satisfied patients are more likely to follow their physicians’ recommendations for treatment and follow-up visits, resulting in better health outcomes and hospital recommendations to others [35].

### 2.2. Social Media Data and Machine Learning

Social media data are often massive and present several difficulties, including data cleansing, data processing, and developing a theoretical model of social media content quality. While this may be accomplished manually via human input, the procedure is time consuming, labour intensive, and the validity and reliability of the technique are often questioned [29]. A comprehensive analysis of POR established and recommended the use of advanced analytical methods such as machine learning to accelerate the processing of huge amounts of online review data [16]. Additionally, the systematic review recommended doing an in-depth examination of the contents of online reviews rather than just comparing structured data to social media ratings. Monitoring service quality through hospital social media platforms may assist all stakeholders in detecting quality issues and minimising the need for expensive and time-consuming surveys. Despite their rarity, research on Facebook content analysis demonstrates a correlation between social media quality domains and traditional hospital quality metrics [23,28,43,44].

The word “themes” or “text classification” refers to the process of grouping together a collection of textual messages according on their content. Machine learning enables automatic topic analysis via the application of various algorithms that are classified as supervised and unsupervised learning. The existence of labels in the subset of training data distinguishes these two main categories [45]. Along with input features, supervised machine learning makes use of predefined output features. The algorithms attempt to forecast and classify the predefined feature, and their accuracy and misclassification, as well as other performance metrics, are determined by the counts of the predetermined feature that are correctly predicted or classified, or that are incorrectly predicted or classified. Manual classification is a technique that is often used in supervised learning. Numerous studies have utilised this approach to deduce the topics of contention in POR [11,12,28,46,47,48].

On the other hand, unsupervised learning is pattern recognition that does not need the usage of a target feature. Unsupervised algorithms identify unlabeled data’s underlying groupings and then label each value. Topic modelling is a technique for automatically identifying topics within a given remark, with the most often used approach being Latent Dirichlet Allocation (LDA). Numerous studies have utilised the technique to elicit information on the themes or subjects of discussion in POR [49,50,51,52,53,54].

According to prior research, POR often addressed issues such as appointment scheduling, wait times, the efficiency of the healthcare system, and interpersonal quality [12,28,46,50]. However, other topics such as communication, technological elements, treatment effectiveness, patient safety, environment, and hospital expenses were recognised as significant concerns [13,38,52,53]. Further study of hospitals in the United States revealed that the variables most significantly linked with patients’ overall ratings or satisfaction included waiting times, treatment effectiveness, communication, diagnostic quality, environmental cleanliness, and economic concerns [28]. Comparable research utilising the Consumer Assessment of Healthcare Providers and Systems (CAHPS) Dental Plan Survey [55] and Press Ganey [56] corroborated the result. Other research discovered that the issues discussed in the dissatisfaction survey mirrored the often-discussed topics of appointment access and wait time [46]. Additionally, patient discontent was often related to personnel, punctuality, and diagnostic problems, while satisfaction was significantly related to interpersonal and technical brilliance [52]. However, Yelp review research discovered that patient satisfaction was related to interpersonal quality of surgical care, while dissatisfaction was related to insurance, billing, and the cost of the hospital visit [50]. Another study examined National Health Service (NHS) tweets using the SERVQUAL model and found that the aspects of responsiveness and assurance were often addressed in negative narratives, while empathy was completely positive [53]. It is unsurprising that some subjects elicited more negative annotations than others, particularly comments about time, money, or pain, which are unlikely to be related to patient satisfaction [12].

### 2.3. Proposed Work

Given the exponential growth of social media in Malaysia and Southeast Asia, it is critical to use technology to improve healthcare services. Meanwhile, although Facebook is a popular social media platform, there has been very little study on machine learning and quality measures using Facebook data [28,57,58]. Given Facebook’s popularity in Malaysia and its growing usage in healthcare, this research seeks to fill a void by investigating whether patient comments in Facebook Reviews can be categorised into SERVQUAL topics, and determining their association with patient satisfaction.

Additionally, this research used supervised machine learning to classify topics. Conventional patient satisfaction surveys have several disadvantages, and social media has been proposed as a potential substitute for evaluating patient satisfaction and mood in real time. According to a systematic review of the use of natural language processing (NLP) and machine learning (ML) to process and analyse patient experience data, manual classification of free text comments remains the ‘gold standard’ method of analysis and is currently the only way to ensure that all pertinent patient comments are coded and analysed [29]. Additionally, the analysis showed that patient inputs produced via free-text supplements to structured questionnaires such as SERVQUAL and HCAHPS were stable in nature, making them an appealing source of data for supervised learning. Numerous studies have utilised supervised machine learning to categorise POR themes [28,47,48,57,59,60,61]. Moreover, we suggested that SERVQUAL dimensions be used to train our machine learning topic classifier. Previous research has classified themes or subjects in POR using structured patient questionnaires such as SERVQUAL [53,62], CAHPS Dental Plan Survey [55] and HCAHPS [50]. The potential results may be compared with those obtained via traditional surveys of patient satisfaction or treatment quality.

Nevertheless, the current body of evidence is still limited, owing to a scarcity of sophisticated statistical studies linking patient satisfaction or hospital quality indicators. A systematic review suggested that more empirical research on POR be conducted using pertinent hypotheses, rigorous design, and data analytics [16]. Thus, this study should go beyond basic descriptive analysis and include the testing of theory-based hypotheses to offer additional policy implications and understanding. Previously published research has utilised analysis of variance (ANOVA) [55], various regression analytical tests [12,52,54,58], Pearson correlation [50,57] or Spearman’s rank correlation [57,63]. As such, this research seeks to examine variables related with patient dissatisfaction using rigorous statistical techniques such as regression analysis.

## 3. Materials and Methods

This research was cross-sectional in design and took place between March 2020 and May 2021. To achieve an equilibrium between subject homogeneity and generalizability of the findings, this research comprised only government hospitals. Universal sampling was utilsed as the sample technique.

### 3.1. Facebook Data

WebHarvy Scraping Software (SysNucleus, Kochi, India) was used to gather data on Facebook reviews from the official Facebook pages of public hospitals in Malaysia from January 2017 to December 2019. First, via the Ministry of Health official website, any webpage link of a public hospital website was sought to be identified. Then a link to the hospital’s official Facebook page inside the hospital’s web page was sought. If there was no link to the hospital’s official Facebook page on the hospital’s website, the search was continued on the Facebook platform. When an official hospital Facebook page was discovered, the information was confirmed by utilising the hospital’s official website’s URL, contacting hospital officials, or using this study’s operational definition for a legitimate hospital Facebook page. An ‘official hospital Facebook page’ was defined as one with a ‘verified tick’ [64] or one with the hospital’s official name (RASMI in the Malay language) included in the Facebook page’s name or in the description of the site. All data gathered from the official Facebook page was kept in a pro forma checklist. The Facebook accounts of hospital departments, health institutions/agencies (such as the Ministry of Health (MOH) or the Institute of Medical Research), non-governmental organizations (NGOs) and long-term care facilities were omitted. These methods of searching have also been used in previous studies [23,24,64]. Malaysia is a multilingual country with a rich variety of languages and dialects. Malay is the national language, while English is the second language. Therefore, reviews were gathered in only those languages. To guarantee that the data language was appropriate and standardised for analysis, a group of junior doctors examined and corrected any spelling and grammatical errors in online reviews written in Malay and English. Then, data in Malay language were manually translated into English for further research by junior doctors. All data were kept in a local database that was encrypted and accessible only to the research team.

### 3.2. Machine Learning Topics Classification

To serve as a “gold standard” for machine learning classifiers, a labeled data set was generated through manual coding. The categorisation was based on the five-dimensional SERVQUAL theoretical notion [8,65]. These categories were: (1) tangible—the appearance of physical facilities, equipment, and healthcare personnel; (2) reliability—the ability to perform the promised services accurately and reliably; (3) responsiveness—the willingness to assist the customer and provide prompt service; (4) assurance—the employee’s knowledge and courtesy, as well as their ability to inspire trust and confidence; and (5) empathy—the ability to empathise with the customer. Two hospital quality managers or SERVQUAL domain experts were assigned to perform initial “open” coding on batches of three hundred Facebook reviews based on the MOH SERVQUAL patient satisfaction survey and other SERVQUAL surveys from previous studies aimed at establishing the source of the coding standard. Intercoder reliability was then determined using a randomly chosen subsample of three hundred Facebook reviews. The raters separately coded the reliability subsample. Inter-rater agreement was determined using Cohen’s Kappa (k) values for each SERVQUAL dimension. The agreement between the coding of tangible (Cohen’s k = 0.885, *p* < 0.001), empathy (Cohen’s k = 0.875, *p* < 0.001), reliability (Cohen’s k = 0.736, *p* < 0.001), and responsiveness (Cohen’s k = 0.72, *p* < 0.001) was high, but the agreement for assurance (Cohen’s k = 0.626, *p* < 0.001) was moderate. Cohen’s k coefficient was 0.769 on average in all dimensions. The machine learning classifier was then trained on a sample of nine hundred manually labelled Facebook reviews.

**Figure 1:**
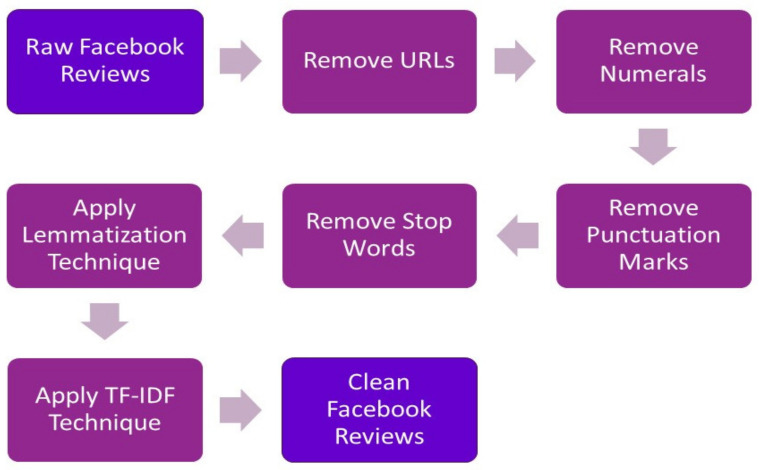
Text Preprocessing using natural language processing (NLP) techniques.

The machine learning technique analysed the characteristics of the individual phrases used in the Facebook reviews, and used this data to build a topic classifier. First, the labeled dataset was pre-processed to remove URLs, numerals, punctuation marks, stop words and simplifying words using a lemmatization technique (e.g., treating as a treat). Following that, the weights of terms were calculated using the term frequency-inverse document frequency (TF-IDF) approach, which demonstrated their significance to the documents and corpus. Figure 1 explains the Natural Language Processing (NLP) techniques used in the text preprocessing phase.

Iterative stratification was used to divide randomly labelled data into 80% for training and 20% for testing. Several multi-label classifier techniques were trained for topic classification, including binary relevance, label powerset, classifier chains, RAkEL (Random k-labELsets), MLkNN (multi-label k-Nearest Neighbor), and BRkNN (Binary Relevance k-NN). For each method, three main classifiers were trained: naive Bayes (NB), support vector machine (SVM), and logistic regression (LR). These classifiers are all widely used methods and have been shown to perform well on text classification tasks [29,31,66]. Multiple label classifiers were evaluated using the scikit-multilearn module in Python [67]. Finally, the various classifiers were evaluated using 5-fold cross-validation.

**Figure 2:**
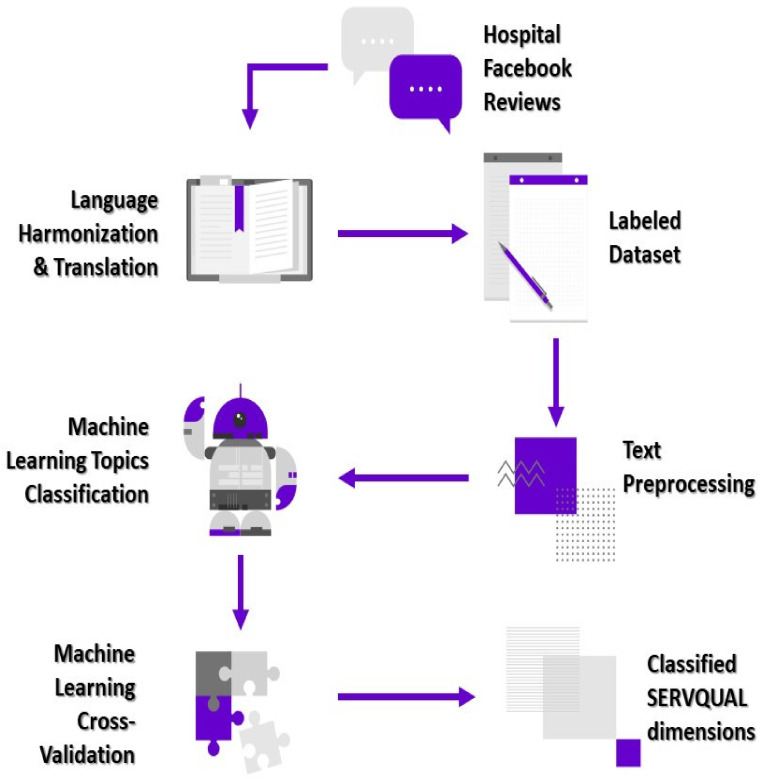
General architecture of proposed methodology in this study.

The 5-fold cross-validation revealed that the machine learning algorithms’ F1-score performance varied between 0.69 and 0.76, suggesting that the models accurately classified the reviews. When different models and classifiers were compared, it was shown that the SVM model with classifier chains multi-label method had the highest accuracy (0.215) and F1-score (0.757). Additionally, the model had the lowest hamming loss (0.273). Hamming loss is a key performance metric in topic classification models since it measures the percentage of erroneous projected class labels. As a consequence, the machine learning classifier was trained using the chains classifier technique on the SVM model. The performance metrics for supervised machine learning with 5-fold cross-validation are summarised in Table 1. The proposed methodology general architecture is depicted in Figure 2.

### 3.3. Outcome: Patient Dissatisfaction

Facebook review is a feature that allows people to leave narrative reviews on organisations’ and companies’ Facebook profiles. Since its debut in 2013, the Facebook review section has been included into the Facebook pages of many hospitals. Patients and their relatives have gradually begun to make use of it. Previously, Facebook utilised a five-star rating system until early 2018, when it switched to a binary rating system named “Recommends” or “Doesn’t Recommend.” This simplified the review process for users. As is the case with other social media platforms, Facebook ratings provide insight on how people feel about healthcare services. Customer recommendations were collected from hospital Facebook pages to determine patient satisfaction. Patient dissatisfaction was characterised as non-recommendation in the Facebook Review section, and patient satisfaction as recommendation. Any recommendation made outside of the Facebook review area was ignored.

### 3.4. Statistical Analysis

Due to the non-normal distribution of the data, medians (interquartile range [IQR]) were used for numerical data, and frequencies and percentages for categorical variables in the statistical analysis. Binary logistic regression analysis was used to evaluate the associations between patient dissatisfaction and multiple factors. Confounding variables included hospital characteristics (region, bed count, urban or rural location, and type of hospital), as well as Facebook page characteristics such as previous star ratings, acceptable hospital information on the Facebook page, and administrator reaction in the Facebook review area. These characteristics, according to previous research, were linked with patient satisfaction [12]. The data were examined to determine whether findings were statistically significant with a *p* value less than 0.05. All statistical tests were verified and found to be valid. Hosmer and Lemeshow tests were used to verify the model fitness, as well as the area under the receiver operating characteristic (ROC) curve. SPSS software version 26 was used to analyse the data (IBM Corp, Armonk, NY, USA).

## 4. Results

### 4.1. Hospital and Facebook Characteristics

In Malaysia, 63.7% of the 135 public hospitals have a Facebook page, with 48 of them accepting customer feedback through Facebook Review. Except for the western part of Malaysia, every region has at least 10 hospitals with a Facebook review function: 37.5% of tertiary hospitals, 8.3% of secondary hospitals, and 54.2% of primary hospitals all have Facebook review sections. The majority of these hospitals are located in cities, with an average of 730 beds. The average number of reviews on each hospital’s Facebook page was 15.5 (27.5), with a previous star rating of 5.00 (1.65).

### 4.2. Facebook Reviews and Patient Satisfaction

A total of 3025 Facebook reviews were collected, with 1200 being used for machine learning training and the rest for association analysis. More Facebook reviews were seen at hospitals in the western (50.5%) and northern (21.5%) areas. Furthermore, urban hospitals accounted for 87.2% of all assessments, tertiary institutions for 88.8%, and the median bed count was 730. The average previous star rating on Facebook in terms of Facebook characteristics was 4.70 (1.5). The majority of Facebook reviews provided sufficient information about the hospital yet received little to no response from hospital management. Most notably, this study discovered that 73.5% were satisfied with the public hospital service, whereas 26.5% were dissatisfied. Table 2 describes hospital Facebook review characteristics.

### 4.3. Classification of SERVQUAL Dimensions

**Figure 3:**
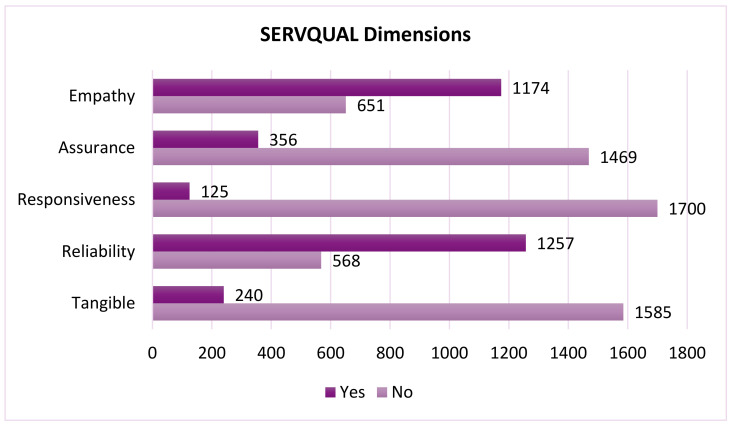
SERVQUAL dimensions classified by machine learning classifier (*n* = 1825).

Using the machine learning topics classification, there were 13.2% reviews with a tangible dimension, 68.9% reviews of reliability, 6.8% reviews of responsiveness, 19.5% reviews of assurance, and 64.3% reviews of empathy. The overall SERVQUAL dimensions are presented in Figure 3.

### 4.4. Factors Associated with Patient Dissatisfaction

To assist MOH and key stakeholders in identifying areas for improvement, binary logistic regression was utilised, with patient dissatisfaction as the primary outcome. When compared with East Malaysia, a univariate study of hospital variables indicated that the three regions were related with patient dissatisfaction: West Coast (Crude OR = 2.11; 95% CI: 1.35–3.30; *p* = 0.001), East Coast (Crude OR = 0.63; 95% CI: 0.41–0.96; *p* = 0.031), and South (Crude OR = 2.38; 95% CI: 1.49–3.80; *p* = 0.001). In addition, patient dissatisfaction was linked to rural hospitals (Crude OR = 1.87; 95% CI: 1.40–2.49; *p* < 0.001) and tertiary hospitals (Crude OR = 0.65; 95% CI: 0.44–0.96; *p* = 0.030). Moreover, a relationship was discovered between previous Facebook star ratings and patient dissatisfaction (Crude OR = 0.86; 95% CI: 0.80–0.93; *p* < 0.001). Reliability (Crude OR = 1.52; 95% CI: 1.20–1.92; *p* = 0.001), responsiveness (Crude OR = 2.10; 95% CI: 1.45–3.04; *p* = 0.001), and empathy (Crude OR = 1.57; 95% CI:1.25–1.97; *p* = 0.001) were all significantly associated with patient dissatisfaction. The univariate study of hospital and Facebook features, as well as SERVQUAL in relation to patient dissatisfaction, is summarised in Table 3.

In multivariate analysis, variables with a *p*-value less than 0.25 in univariate analysis were chosen throughout the model selection phase. Forward LR, backward LR, and manual selection methods were used to create a parsimonious model. The final model included hospital location and SERVQUAL dimensions other than tangible and assurance. When chosen SERVQUAL dimensions were controlled, hospitals situated in rural areas had a 100% higher likelihood of patient dissatisfaction compared with hospitals located in urban areas (95% CI:1.49–2.68; *p* < 0.001). Most importantly, when other variables were adjusted, reliability had a 113% higher likelihood of patient dissatisfaction (95% CI: 1.63–2.78; *p* < 0.001), responsiveness had a 61% higher likelihood of patient dissatisfaction (95% CI:1.09–2.38; *p* = 0.016), and empathy had a 108% higher likelihood of patient dissatisfaction (95% CI:1.63–2.69; *p* < 0.001). There was no interaction and multicollinearity in the multivariate model. The model’s fitness was also satisfactory, as verified by the Hosmer and Lemeshow Test (*p* = 0.875), 73.5% of the classification table, and 61.7% of the area under the receiver operating characteristic (ROC) curve (*p* < 0.001). Table 4 details the multivariate analysis.

## 5. Discussion

POR influences patient preferences, emphasising the critical role of patient-centered health care and changing the system. The research is a critical first step in developing a strategy for utilising social media data in Malaysia, as well as a first effort to monitor public views of healthcare services using a novel data source. This is the first study to use automated computer methods to assess topics from online hospital evaluations and to characterise the content of narrative online hospital reviews in Malaysia. According to the machine learning classifier, the SERVQUAL dimension with the greatest frequency was reliability, followed by empathy. The reliability dimension was often concerned with appointment scheduling, punctuality, the healthcare system’s efficacy, and the capability to keep accurate data.

Meanwhile, the problem of empathy related specifically to staff attention and helpfulness, an understanding of patient requirements, convenient hospital hours, and a commitment to the patient’s best interests. These findings supported previous studies indicating that online reviews often emphasise time promise, healthcare system efficiency, and interpersonal quality [11,12,28,46,50]. However, additional topics were identified in the POR as major concerns, including communication, therapeutic effectiveness and patient safety, the environment, and hospital costs [13,38,52,53]. Moreover, most online patients reported satisfaction with the treatments provided by Malaysian hospitals. The findings supported comprehensive studies of patient online evaluations, which showed that the majority of patients were satisfied with their healthcare providers and would recommend them to family and friends [16,68].

Patient satisfaction surveys assist health care workers in identifying opportunities for service improvement. Additionally, they enable authorities to understand patient needs and create strategic plans for more effective and high-quality services [35]. This study found that hospital characteristics such as location in the western and southern regions, as well as rural locations, were associated with patient dissatisfaction. This was supported by African research [39], despite the fact that an Asian survey found rural residents to be generally satisfied with healthcare services [40]. Additionally, the size and type of hospital services had an effect on patient satisfaction [15,41]. Previously, it was believed that people would be more unhappy with a service that dealt with a greater number of patients and a bigger practice. However, this study found a negative correlation between tertiary centre and patient dissatisfaction, suggesting that patients were pleased with the service given by bigger types of hospitals, owing to the comprehensive healthcare services provided.

Interpersonal skills (empathy) were shown to be a major factor in increased patient satisfaction [35,69,70]. In this study, the empathy component was shown to be positively associated with patient dissatisfaction. The finding was confirmed by a social media study performed in China [13] and research conducted on the NHS Choices website [71], both of which revealed further negative comments regarding the doctor–patient connection, nurse service, roughness, and apathy. Moreover, a comparative study of POR in China and the United States found that the majority of complaints addressed the doctor’s or hospital staff’s bedside demeanour [51]. However, data from NHS Twitter showed that patients expressed a high degree of satisfaction with the empathy component of healthcare [53]. Physicians and nurses were assessed on their interactions with patients and their family or friends, including their friendliness, honesty, concern, compassion, empathy, kindness, civility, and respect for patient preferences [35,70]. Patients who were satisfied with physicians’ affective behaviours were more likely to recommend them to others, according to research performed at a Scottish NHS trust [72].

Another area in which Malaysian public hospitals might improve is their reliability. A positive and statistically significant relationship was found between reliability and patient dissatisfaction in public hospitals. It is unsurprising that the majority of patient complaints or dissatisfaction voiced through POR concerned time commitment, appointment or follow-up access, and service inefficiencies [12,13,28,46,51]. Patient satisfaction was positively linked with ease of access to the hospital, convenient location, a streamlined admission and discharge procedure, and an efficient appointment system [35]. According to one study, scheduling convenience and adequate follow-up may help reduce patient dissatisfaction [54]. Additionally, local research has shown that the “lean” strategy may be effectively utilised to improve hospital reliability [73].

Responsiveness was defined as the willingness of healthcare professionals and providers to assist and give timely service to clients. A positive and statistically significant connection was found between responsiveness and patient dissatisfaction. Similar findings have been reported in earlier local research [74,75] as well as in international SERVQUAL studies [10,76]. Additionally, experimental research of the perceived SERVQUAL model using tweets from the NHS UK found that people expressed their dissatisfaction with responsiveness more than with other elements [53]. Patient satisfaction was shown to be positively linked with reduced wait times and quick treatment in a systematic study [35]. A comprehensive study showed that a wait time of more than 17 min decreased the probability of obtaining a good rating status [54].

Although this research discovered no significant connections between assurance and tangible dimensions with patient dissatisfaction, it is worth highlighting the dimensions’ predictive value in POR. The quality of technical care was closely related to elements of assurance such as human competency, professionalism, and confidentiality [35]. Moreover, it pertained to the services’ compliance with clinical diagnostic and treatment standards and recommendations. Numerous studies have found an association between assurance-related topics and patient satisfaction, including treatment effectiveness, diagnostic quality, and treatment side effects, utilising theme analysis of social media data [28,77]. Meanwhile, a study comparing POR in China and the United States found that both nations’ citizens were dissatisfied with medical treatment [51]. Previously, it was thought that those who felt they had been treated unfairly were less satisfied with health care services. However, since some patients were unable to evaluate the technical quality of therapy due to their limited comprehension, they may have replaced their judgement for the sense of how nice and caring health professionals were toward them [35].

The physical environment was another important factor influencing patient satisfaction. Patient satisfaction was expected to be related to the pleasantness of the environment, cleanliness, noise level, food service, toilet comfort, clarity of signs and instructions, layout of equipment and facilities, and parking. Few studies have shown that patient satisfaction is influenced by attractive facilities, environmental cleanliness, and design-related factors [28,38,40,46]. However, further research showed that patients were unhappy with aspects of the hospital atmosphere based on their online assessments [46,53,61,69]. Malaysia’s government has spent millions of ringgits in a series of Malaysia Plans aimed at enhancing public hospital facilities and services and building new hospitals [78]. As a result, hospital clients appreciate the upgrade and improvement of public hospital assets on social media.

These findings have a number of implications for many aspects of hospital quality of care. To begin, quality-of-care metrics and patient satisfaction can be monitored and evaluated in real time by using hospital Facebook reviews and machine learning algorithms. The method used in this study enables policymakers to make use of social media data rather than more expensive national questionnaire surveys. Moreover, there is no comparable open-standard research of patient satisfaction in Malaysia’s public and private sectors. While the Ministry of Health prefers the SERVQUAL questionnaire, private hospitals may develop their own or adhere to an international standard. As a result, Facebook reviews may serve as a new barometer of patient satisfaction in each of these domains. Additionally, Facebook reviews are straightforward and accessible, reducing obstacles to obtaining information about hospital quality and helping hospitals in addressing quality-of-service problems while also alerting hospitals to possible patient safety concerns. While social media ratings are untested and unregulated, traditional patient satisfaction surveys have been validated and tested. By including additional hospital quality metrics on hospital Facebook pages and critical information such as the official status of the Facebook site and the exact Facebook addresses, the validity of Facebook data will be increased [23].

Furthermore, this research has highlighted three SERVQUAL characteristics, namely reliability, responsiveness, and empathy, that need additional attention and improvement on the part of Malaysian healthcare authorities. Enhancing interpersonal skills training, especially for medical students, ongoing training for health professionals in the workplace, and lean model adaption will substantially enhance the quality of treatment that is currently lacking [79,80]. However, health authorities must realise that the findings are unlikely to be representative of the whole population served by hospitals. Rather than that, this study of service quality issues should be seen as a complement to more traditional data collection efforts and as an effective early warning system for hospital quality management.

### Future Works and Limitations

Future study should concentrate on improving the efficacy of machine learning classifiers and collecting a bigger dataset of POR, including those from the Malaysian private sector. Second, further research is required to establish the relationship between POR and other hospital quality or clinical outcome measures, as earlier studies have done [11,12,43,63,81]. Additionally, future research may incorporate additional social media platforms (e.g., Twitter, Instagram, Tik-Tok, etc.) with specific adjustments such as a focus on the youth population (targeted audience), common public health topics discussed on social media platforms (depression, vaccination, cyberbullying, etc.), as well as identifying popular hashtags related to public health issues. The data collected from various social media platforms may offer healthcare agencies with a unique viewpoint on patients and may be utilised as a real-time public health surveillance system.

This research has a number of limitations. Due to the cross-sectional nature of the research, the possibility of a causal connection in our findings cannot be ruled out. Moreover, almost one-third of public hospitals posted feedback on Facebook. Incorporating unauthorised Facebook pages for public hospitals may have a contrasting impact. Additionally, the research dataset is considered small-scale in comparison to other POR research, due to Malaysia’s small population and the relatively recent adoption of POR in the Malaysian healthcare sector. Malaysians, on the other hand, have a high rate of internet usage, which continues to grow year after year, thus a surge of POR about healthcare services may be expected over the next few years. Additionally, the main limitation was the time needed for content analysis and manual coding. Comprehensive reading and classification of datasets remains the gold standard for building machine learning-based topic classifiers and is the only way to ensure that all essential comments are coded [29]. However, it is time consuming, and in text classification, increasing the diversity of comments lowers the ability of the machine learning system to properly recognise the remark. However, if social media input becomes more prevalent, manual coding may become problematic owing to time constraints, and topic modelling may be a viable alternative. Topic modelling using Latent Dirichlet Allocation (LDA) may aid in determining how well the results fit the themes chosen by domain experts, and this unsupervised approach will allow the identification of previously undiscovered topics [82].

## 6. Conclusions

Patient online reviews offer healthcare authorities a practical, low-cost, and accessible way of collecting information about the quality of care they deliver. Healthcare officials have long considered how to include POR into citizen-government engagement and policymaking in order to create evidence-based reporting. Despite scholars’ focus on the potential for POR data to assist in decision making, methods for realising this potential have been very restricted, often fragmentary, and non-standardised. This research suggested a systematic method for integrating POR data in order to analyse and monitor patient perceptions of the service quality at Malaysian public hospitals. Automatically classifying Facebook reviews into SERVQUAL dimensions using machine learning minimised human interference and selection bias in the study. Classification performance was verified, with an emphasis on the criticality of collecting reliable quality of care topic sets using the SERVQUAL model, and used to grasp the context of Facebook reviews. Despite the fact that the majority of POR were found to be satisfied with the hospital service, this study highlighted SERVQUAL dimensions of reliability, responsiveness, and empathy as areas for quality-of-care improvement in Malaysian public hospitals. Additionally, public hospital service in rural areas was associated with patient dissatisfaction. The results provide important insights that will aid healthcare officials and authorities in capitalising on the opportunities of POR by monitoring and assessing services’ quality in order to make rapid improvements. Furthermore, the findings of traditional patient satisfaction surveys may be routinely supplemented with data from POR to continually improve and create high-quality healthcare services.

## Figures and Tables

**Table 1:** Overall ML models performance with 5-fold cross-validation.

Multilabel Classifier	Model	Accuracy	Recall	Precision	F1-Score	Hamming Loss
Binary Relevance	NB	0.147	0.761	0.701	0.730	0.315
	SVM	0.211	0.763	0.745	0.754	0.278
	LR	0.193	0.775	0.732	0.753	0.285
Label Powerset	NB	0.130	0.896	0.633	0.741	0.349
	SVM	0.166	0.799	0.679	0.734	0.323
	LR	0.158	0.825	0.669	0.739	0.326
Chains Classifier	NB	0.149	0.756	0.705	0.730	0.313
	SVM	0.215	0.761	0.753	0.757	0.273
	LR	0.191	0.770	0.727	0.748	0.290
RAkEL	NB	0.157	0.749	0.699	0.722	0.322
	SVM	0.186	0.764	0.724	0.743	0.295
	LR	0.180	0.765	0.726	0.745	0.293
MLkNN	N/A	0.140	0.737	0.697	0.715	0.327
BRkNN	N/A	0.157	0.648	0.732	0.687	0.330

NB, naive Bayes; SVM, support vector machine; LR, logistic regression.

**Table 2:** Hospital Facebook review characteristics (*n* = 1825).

Variable		*n*	(%)	Median	(IQR)
*Hospital Features*					
Region	East Coast	189	(10.4)		
	North	393	(21.5)		
	West	922	(50.5)		
	South	178	(9.8)		
	East Malaysia	143	(7.8)		
Location	Rural	234	(12.8)		
	Urban	1591	(87.2)		
Hospital Type	Primary	125	(6.8)		
	Secondary	80	(4.4)		
	Tertiary	1620	(88.8)		
Beds				730	(563)
*Facebook Features*					
Previous Facebook Star Ratings				4.70	(1.5)
Admin Response	No	1651	(90.5)		
	Yes	174	(9.5)		
Adequate Hospital Information	No	1651	(90.5)		
	Yes	174	(9.5)		
Patient Satisfaction	Dissatisfied	483	(26.5)		
	Satisfied	1342	(73.5)		

**Table 3:** Factors associated with patient dissatisfaction in univariate analysis (*n* = 1825).

Variables		Crude OR	95% CI	*p*-Value *
			(Lower, Upper)	
*Hospital Features*				
Region	East Malaysia	Ref		
	East Coast	0.63	0.41, 0.96	0.031
	North	1.08	0.75, 1.55	0.695
	West	2.11	1.35, 3.30	0.001
	South	2.38	1.49, 3.80	<0.001
Location	Urban	Ref		
	Rural	1.87	1.40, 2.49	<0.001
Hospital Type	Primary	Ref		
	Secondary	0.97	0.54, 1.76	0.924
	Tertiary	0.65	0.44, 0.96	0.030
Beds		1.00	1.00, 1.00	0.275
*Facebook Features*				
Admin Response to Review	No	Ref		
	Yes	1.24	0.88, 1.75	0.210
Adequate Hosp Info	No	Ref		
	Yes	0.80	0.53, 1.22	0.306
Facebook Star Ratings		0.86	0.80, 0.93	<0.001
*SERVQUAL*				
Tangible	No	Ref		
	Yes	1.25	0.93, 1.69	0.137
Reliability	No	Ref		
	Yes	1.52	1.20, 1.92	0.001
Responsiveness	No	Ref		
	Yes	2.10	1.45, 3.04	<0.001
Assurance	No	Ref		
	Yes	0.96	0.74, 1.25	0.766
Empathy	No	Ref		
	Yes	1.57	1.25, 1.97	<0.001

* Simple logistic regression.

**Table 4:** Factors associated with patient dissatisfaction in multivariable analysis (*n* = 1825).

Variable		Adjusted	Adjusted 95% CI	*p*-Value *
		OR	(Lower, Upper)	
Location	Urban	Ref		
	Rural	2.00	1.49, 2.68	<0.001
Reliability	No	Ref		
	Yes	2.13	1.63, 2.78	<0.001
Responsive	No	Ref		
	Yes	1.61	1.09, 2.38	0.016
Empathy	No	Ref		
	Yes	2.08	1.61, 2.69	<0.001

* Multiple logistic regression, constant = −2.180, forward LR, backward LR and manual selection methods were applied, no significant interaction or multicollinearity. Hosmer and Lemeshow test = 0.875, classification table = 73.5%, area under the operating curve (ROC) = 61.7% (*p* < 0.001).

## Data Availability

The Facebook data presented in this study are available on request from the corresponding author. The data are not publicly available due to privacy.
